# Toads phenotypically adjust their chemical defences to anthropogenic habitat change

**DOI:** 10.1038/s41598-019-39587-3

**Published:** 2019-02-28

**Authors:** Veronika Bókony, Bálint Üveges, Viktória Verebélyi, Nikolett Ujhegyi, Ágnes M. Móricz

**Affiliations:** 1 Lendület Evolutionary Ecology Research Group, Plant Protection Institute, Centre for Agricultural Research, Hungarian Academy of Sciences, Herman Ottó út 15, 1022 Budapest, Hungary; 2 Institute for Biology, University of Veterinary Medicine, Rottenbiller u. 50, 1077 Budapest, Hungary; 3 Department of Pathophysiology, Plant Protection Institute, Centre for Agricultural Research, Hungarian Academy of Sciences, Herman Ottó út 15, 1022 Budapest, Hungary

## Abstract

Despite the well-documented effects of human-induced environmental changes on the morphology, physiology, behaviour and life history of wild animals, next to nothing is known about how anthropogenic habitats influence anti-predatory chemical defence, a crucial fitness component of many species. We investigated the amount and composition of defensive toxins in adult common toads (*Bufo bufo*) captured in natural, agricultural and urban habitats, and in their offspring raised in a common-garden experiment. We found that, compared to toads captured from natural habitats, adults from both types of anthropogenic habitats had larger toxin glands (parotoids) and their toxin secretion contained higher concentrations of bufagenins, the more potent class of bufadienolide toxins. Furthermore, urban toads had lower concentrations of bufotoxins, the compounds with lower toxicity. None of these differences were present in the captive-raised juveniles; instead, toadlets originating from agricultural habitats had smaller parotoids and lower bufotoxin concentrations. These results suggest that toads’ chemical defences respond to the challenges of anthropogenic environments via phenotypic plasticity. These responses may constitute non-adaptive consequences of pollution by endocrine-disrupting chemicals as well as adaptive adjustments to the altered predator assemblages of urban and agricultural habitats.

## Introduction

Natural habitats worldwide are increasingly modified by human-induced environmental change; for example, agricultural and urban areas have taken up about 40% and 5% of the Earth’s land surface, respectively^1,2^. The abiotic and biotic changes induced by both forms of anthropogenic land use include habitat fragmentation, various kinds of pollution, and altered flora and fauna with lower diversity and more invasive species^3–5^. Over the past two decades, a surge of studies documented wide-ranging effects of these environmental changes on the morphology, physiology, behaviour and life history of wild organisms^3–8^. Some of these phenotypic changes are maladaptive and contribute to population declines, while some are adaptive and help living in anthropogenic habitats^4^. The adaptive responses can result from phenotypic plasticity of individuals or from genetic divergence between populations^3–5^. In most cases, there is yet insufficient knowledge to identify which of these two mechanisms are responsible, although such knowledge would be crucial for predicting and dealing with the eco-evolutionary consequences of anthropogenic environmental change^3–5^.

Animals’ chemical defences are especially under-studied in this context. Like plants, many animal species rely on defensive chemicals or toxins for protection from their natural enemies such as predators, parasites and competitors^9^. Chemical defence may have important consequences for life-history evolution and ecology, as chemically protected animals can live longer^10^ and occupy a larger niche space^11^. Changes in toxicity can affect not only the defended animal’s survival^12^ but also other species’; for example, predators can suffer serious mortality when consuming unusually toxic prey which can lead to predator population declines^13^. Some predators can learn to avoid toxic prey and switch to other species which then may alter trophic interactions and community structure^14,15^; whereas other predators adapt to consuming toxic prey by evolving toxin resistance, leading to co-evolutionary arms races between the defended organisms and their enemies^16^. Despite this potential of defensive toxins to impact multiple populations across wildlife communities and thereby biodiversity conservation, we have very little understanding of how environmental changes influence chemical defences in animals^9,17^, and we know virtually nothing about whether and how their toxicity is altered by anthropogenic habitats.

**Figure 1:**
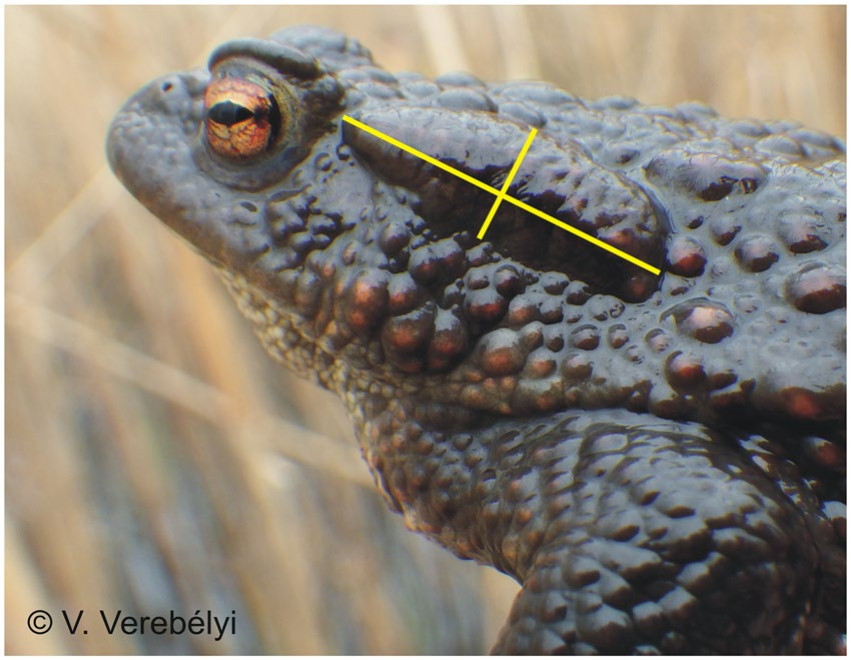
An adult common toad, with the length and width of the left parotoid gland shown by yellow lines.

In this study, we investigated the chemical defences of common toads (*Bufo bufo*) in natural and anthropogenically influenced environments. Bufonid toads synthesize toxic steroids called bufadienolides, which they store in their skin glands including their main toxin depot, the pair of parotoid glands^13^ (Fig. 1). These toxic compounds are potent inhibitors of Na^+^/K^+^-ATPase activity, causing upon ingestion a bitter taste, nausea or heart failure^18–20^, and thereby they often repel or can even kill predators^13,21^. Bufadienolides may also contribute to the toads’ immune defence against pathogens^22^. Bufagenins, the smaller, hydrolysed bufadienolide molecules usually have stronger cardiotoxic effects than bufotoxins, the larger bufadienolide molecules with an amino-acid side chain^19,23^. Common toads start to produce both types of bufadienolides as young tadpoles^24^, and they flexibly adjust their toxin levels to larval environmental conditions like food availability^24^ and competitor density^25^. Recent experiments also showed that chronic exposure to an agricultural pollutant increased the bufadienolide content of common toad tadpoles^26^. Because anthropogenic habitats are characterized by higher levels of chemical pollution^27^ and differ from natural habitats in many further environmental factors^3–8^ that may also affect the animals’ ability and/or need to defend themselves with toxins, we hypothesized that adult toads may have altered toxin levels in agricultural and urban habitats. To test this idea, we captured free-living adult toads in different habitats and assessed two aspects of their chemical defence: parotoid size as proxy for the total amount of toxins^12^ and the chemical composition of their parotoid secretions. Then, to infer whether the differences we observed between toads from natural and anthropogenic habitats were due to microevolution or phenotypic plasticity, we used a common garden experiment to compare the chemical defences of juveniles raised from the eggs of the adults captured from different habitats.

## Methods

### Data collection

We captured adult toads from 9 ponds in Hungary, which were located in natural, urban or agricultural habitats, with 3 ponds per habitat type^27^. The capture sites are described in detail in the Supplementary Material, including geoinformatics measurements of land use that verified our habitat categorization (Supplementary Methods 1.1, Table S1, Fig. S1). We hand-collected toads at each pond at the start of the spawning season, between 16 and 28 March 2017. We transported the captured animals to our laboratory in Budapest, where they were kept until spawning as described in an earlier paper^27^ (see Supplementary Methods 1.2). After spawning, we measured each toad’s snout-vent length (SVL) and the length and width of the left parotoid^28,29^ to the nearest 0.1 mm with a calliper (Fig. 1). We obtained a toxin sample from each toad by pressing the right parotoid gland and wiping off the secretion with a cotton swab (in 4 animals, both parotoids had to be sampled to get enough secretion). The swab was immediately placed into a microcentrifuge tube filled with 1 mL HPLC-grade absolute methanol. Our sample size was 166 adult toads (72, 52, and 42 from natural, urban, and agricultural habitats, respectively); the parotoid of one agricultural female could not be measured because it was scarred and deformed.

From the eggs laid by the toads in our lab, we raised toadlets as described earlier^27^ (see Supplementary Methods 1.2) until they reached ca. 5 months of age after metamorphosis. At this age they are large enough (ca. 4 g) for measuring parotoid size (ca. 7.5 mm long and 2.5 mm wide) and their gonads are developed enough for sex identification. We photographed each juvenile in a standardized setting to measure their SVL and parotoid size (Supplementary Methods 1.3, Table S2, Fig. S2). After euthanizing the toadlets using a water bath of 5.4 g/L MS-222 buffered with the same amount of Na_2_HPO_4_ to neutral pH^30,31^, we determined their sex by inspecting the gonads, and we preserved their bodies in 96% ethanol. Using the preserved specimens, we measured the length and width of the left parotoid to the nearest 0.1 mm with a calliper; then we obtained a toxin sample from each juvenile by cutting out a sample of the parotoid (a small piece of tissue that constituted a major part of the gland) and we stored it in 1 mL HPLC-grade absolute methanol. We used somewhat different methods for the juveniles than for the adults because the toadlets were raised, photographed and euthanized as part of another experiment, and we decided to study their chemical defences only after finishing the analyses of adult data, when the toadlets had already been sacrificed. For the purposes of the present study, we used one offspring of each pair; 73 juveniles in total (35, 23, and 15 from natural, urban, and agricultural origin, respectively; sample sizes are lower for juveniles than for adult pairs because some pairs refused to spawn in the lab and a few offspring died before sampling^27^).

We measured the amount of bufadienolide compounds in the samples by high-performance liquid chromatography and mass spectrometry following established protocols^24–26,32^, with slight modification as detailed in the Supplementary Material (Supplementary Methods 1.4, Table S3). We captured and handled the animals in accordance with the permits issued by the Government Agency of Pest County (Department of Environmental Protection and Nature Conservation) and the Budapest Metropolitan Municipality (Department of City Administration, FPH061/2472-4/2017). The study was further approved by the Ethical Commission of the Plant Protection Institute, Centre for Agricultural Research, Hungarian Academy of Sciences.

### Statistical analyses

All analyses were run in the R 3.5.0 environment^33^, using the packages nlme^34^ and metafor^35^. First we tested if site of origin (i.e. pond within habitat type) was a significant random effect, because toads from one site may be non-independent from each other^36^. For all variables we found that site of origin as a random effect did not improve model fit significantly (Supplementary Results 2.1, Table S4), so we did not include it in subsequent analyses (i.e. we treated all animals from one habitat type as one sample of independent data, regardless of the specific site they originated from)^36^.

As proxy for parotoid size, we estimated the base area of the left parotoid gland from its length and width by assuming the shape of an ellipse^28^ (Fig. 1, Fig. S2). To test whether toads originating from urban and agricultural habitats differed from toads originating from natural habitats in their parotoid size, we used linear models with parotoid size as the dependent variable, habitat type as a fixed factor, and SVL as a covariate. We also added sex as a fixed effect and the interaction between sex and SVL to allow for the relationship between SVL and parotoid size to have different slope in males and females. This was needed because adult females were significantly larger (SVL: 72.3–118.0 mm, mean ± SE: 100.2 ± 0.74) than adult males (SVL: 67.7–88.5 mm, mean ± SE: 77.9 ± 0.72; Welch’s t-test: t_114.9_ = 21.3, P < 0.001), although there was no sexual dimorphism in juveniles’ SVL (males: 27.5–38.7 mm, mean ± SE: 34.85 ± 0.34, females: 28.5–38.5 mm, mean ± SE: 34.45 ± 0.39, Welch’s t-test: t_114.9_ = 0.76, P = 0.448). As we had two alternative measurements for parotoid size in juveniles, we present the results on calliper measurements (i.e. the method that was used in adults) in the main text and photo measurements in the Supplementary Material (Supplementary Results 2.2, Table S6; see also Supplementary Methods 1.3, Table S2).

To analyse toxin composition, we estimated the concentration of each compound in the parotoid secretion as the marinobufotoxin-equivalent quantity of each bufadienolide compound per 1 mg dry mass of toxin sample (Supplementary Methods 1.4). First we analysed all compounds in a single linear model for each age group, which showed that the differences between habitat types varied significantly among compounds (Supplementary Results 2.1, Table S5). This means that the effect of habitat depends on the compound (and also on sex in adults; Table S5). Thus, the habitat effect cannot be efficiently tested with commonly used statistics like t-tests for each compound or post-hoc tests from a linear model, because the large number of tests would seriously inflate type-1 error. Therefore we used within-study meta-analysis^37^ to estimate the overall effect of habitat type on bufadienolide concentrations and to identify moderator variables that influence the habitat effects. Meta-analysis is an efficient approach that maximizes statistical power and promotes the interpretation of results in terms of effect sizes as is desirable in ecology and evolutionary biology^37^.

As the first step of our meta-analysis, we calculated effect sizes as Hedges’ d (unbiased Hedges’ g)^38^ for the differences of each compound’s concentration between the animals originating from natural habitats and the animals originating from either urban or agricultural habitats (i.e. one urban and one agricultural effect size for each compound). Hedges’ d is a standardized difference in means (i.e. difference between the means of two groups divided by their pooled and weighted standard deviation, multiplied by a correction factor for sample size)^38^. For the adults, we calculated the effect sizes for each compound for the two sexes separately, because 6 out of 31 compounds were not detected in the majority (86–100%) of males (we did not calculate effect sizes for the latter 6 compounds of males; see also Table S5). Furthermore, we omitted one compound because it was not detected in 84% of adults (99% of males and 69.1% of females; bufagenin 7 in Table S3). Thus, we had 108 effect sizes for the adults. In the juveniles, all detected compounds were found in the majority of individuals (Table S3) and there were no sex differences in bufadienolide concentrations (Table S5), so we calculated the urban and agricultural effect sizes for all toadlets regardless of sex, resulting in 40 effect sizes for 20 compounds. Note that this approach has the further benefit of keeping the sample sizes for effect-size estimation relatively constant, as we had toxin data for ca. twice as many adults (n = 166) as juveniles (n = 73). The distribution of raw data used for calculating effect sizes are shown in Fig. S3.

In the second step, we analysed the effect size estimates (i.e. standardized difference between natural and anthropogenic habitats for each compound) in meta-analysis models, which are similar to weighted regression analyses, i.e. they account for the uncertainty of estimates^37^. We conducted separate meta-analyses for adults and juveniles. In each meta-analysis, compound was included as a random intercept to take into account the non-independence of effect sizes comparing the data of urban and agricultural animals to the same animals of natural origin for any given compound. For both age groups, we ran 3 meta-analyses. The first was a simple meta-analytical model (an intercept-only model without any moderators) on all available data to examine the overall effect of anthropogenic habitats on the concentrations of bufadienolides. In the second model we added habitat type as a moderator to test if urban and agricultural habitats had different effects. In the third model we added the interaction of habitat type and toxin type to test if the effects of each habitat type differed between bufotoxins and bufagenins. Point estimates from statistical models were considered significantly different from zero when their 95% confidence intervals (CI) did not overlap zero.

## Results

### Toxin gland size

**Figure 2:**
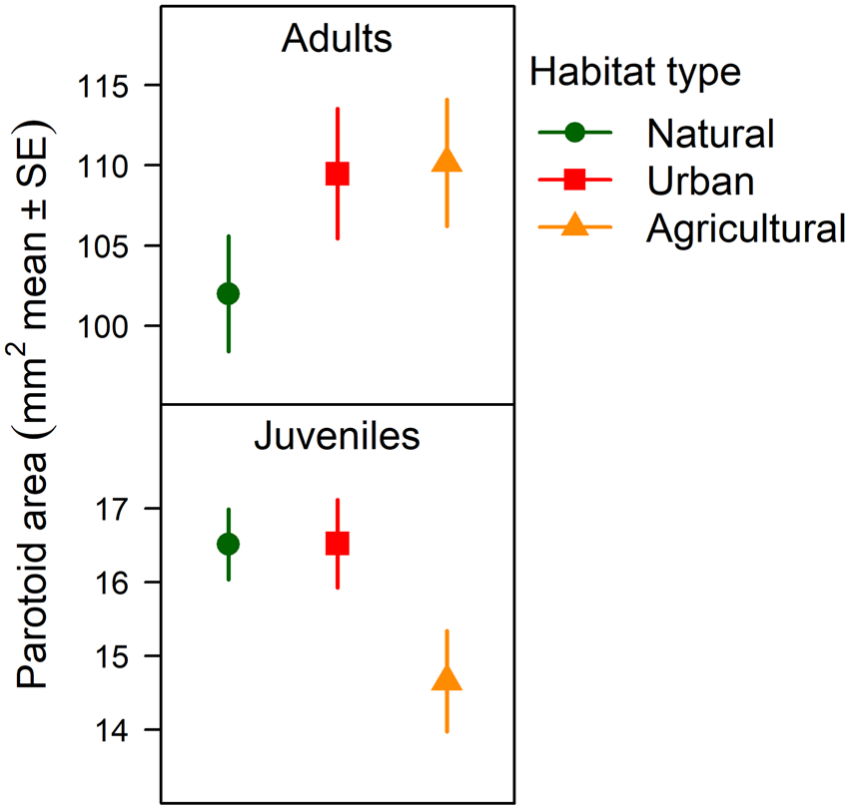
Parotoid size in wild-caught adult toads and their captive-reared offspring, as estimated by the models in Table 1.

In adults, parotoid area was significantly larger in animals captured from urban as well as agricultural habitats compared to toads from natural habitats (Table 1, Fig. 2). In contrast, juveniles originating from agricultural habitats had significantly smaller parotoids than juveniles from natural habitats (Table 1, Fig. 2, Table S6), while toadlets from urban habitats had similar parotoid size as toadlets from natural habitats (Table 1, Fig. 2, Table S6).

**Table 1:** Parameter estimates of linear models for parotoid size (measured with calliper in mm^2^) in 165 adults and 73 juveniles.

Age group	Model parameters	Estimate	SE	t	P
Adults	Intercept (natural, male)	95.229	5.989	15.90	<0.001
	SVL (mm, mean-centered)	1.332	0.482	2.76	0.006
	Sex (female)	14.030	6.642	2.11	0.036
	**Habitat (agricultural)**	**8**.**161**	**3**.**664**	**2**.**23**	**0**.**027**
	**Habitat (urban)**	**7**.**491**	**3**.**349**	**2**.**24**	**0**.**027**
	Sex × SVL	1.447	0.536	2.70	0.008
Juveniles	Intercept (natural, male)	16.154	0.561	28.78	<0.001
	SVL (mm, mean-centered)	0.749	0.212	3.54	0.001
	Sex (female)	0.648	0.630	1.03	0.307
	**Habitat (agricultural)**	**−1**.**854**	**0**.**850**	**−2**.**18**	**0**.**033**
	Habitat (urban)	0.012	0.802	0.02	0.988
	Sex × SVL	−0.193	0.270	−0.71	0.478

Significant habitat effects are highlighted in bold.

### Toxin composition

**Figure 3:**
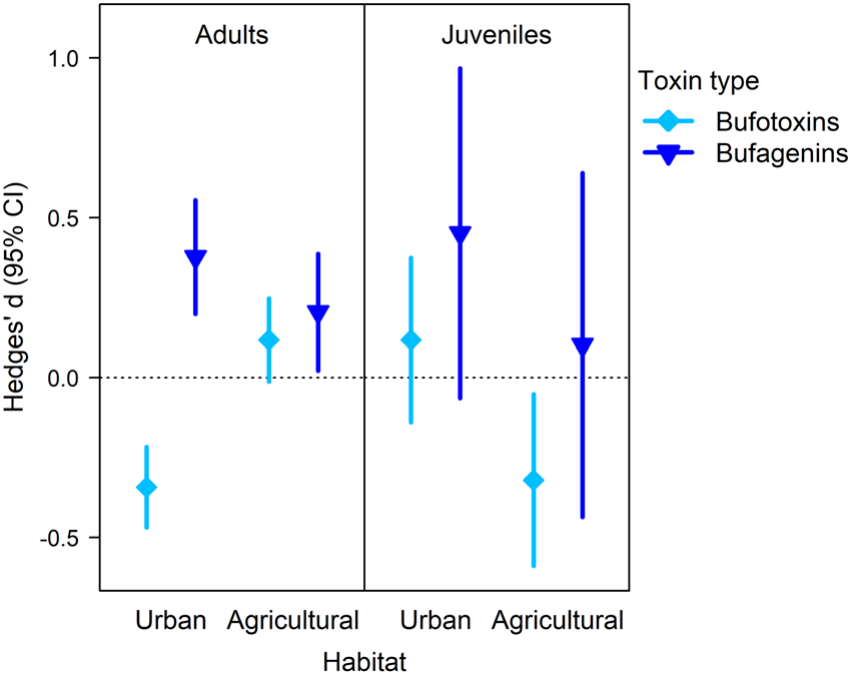
Effects of anthropogenic habitats on toxin composition in wild-caught adult toads and their captive-reared offspring. Meta-analytic means with 95% confidence intervals (CI) express the standardized differences between natural and anthropogenic habitats in bufadienolide concentrations (amount of each compound per unit dry mass of toxin sample). Thus, a positive value means larger concentration of the given type of bufadienolides in animals from the anthropogenic habitat than in animals from the natural habitat.

For adults, the meta-analytic mean effect of anthropogenic habitats on the concentrations of bufadienolide compounds did not differ from zero (Table 2), but the effect sizes varied significantly by habitat type (moderator effect of habitat type, agricultural compared to urban: 0.25 ± 0.05, P < 0.001). Compared to the toxin samples of adults from natural habitats, the overall concentration of bufadienolides was significantly higher in agricultural samples (Table 2) and lower in urban samples, although the latter effect was not quite significant (Table 2). The interaction of habitat type and toxin type was also significant (P < 0.001): compared to toads from natural habitats, toads from both anthropogenic habitats had more bufagenins (Table 2, Fig. 3), but while urban toads had less bufotoxins (Table 2, Fig. 3), toads from agricultural habitats had slightly (although not significantly) more bufotoxins per unit dry mass of parotoid secretion (Table 2, Fig. 3).

**Table 2:** Results of meta-analyses of toxin composition in wild-caught adult toads and their captive-reared offspring. Meta-analytic means (Hedges’ d) with 95% confidence intervals (CI; in brackets) represent the standardized differences between natural and anthropogenic habitats in bufadienolide concentrations (amount of each compound per unit dry mass of toxin sample).

Meta-analysis	Effect	Adults	Juveniles
Model 1	Anthropogenic habitats	0.013 (−0.102, 0.127)	0.005 (−0.215, 0.225)
Model 2	Urban habitats	−0.106 (−0.231, 0.019)	0.184 (−0.050, 0.418)
	Agricultural habitats	**0**.**144 (0**.**018**, **0**.**271)**	−0.236 (−0.481, 0.008)
Model 3	Urban habitats		
	Bufotoxins	**−0**.**343 (−0**.**469**, **−0**.**217)**	0.117 (−0.140, 0.374)
	Bufagenins	**0**.**377 (0**.**198**, **0**.**555)**	0.451 (−0.064, 0.967)
	Agricultural habitats		
	Bufotoxins	0.117 (−0.012, 0.246)	**−0**.**321 (−0**.**590**, **−0**.**052)**
	Bufagenins	**0**.**204 (0**.**022**, **0**.**387)**	0.101 (−0.437, 0.639)

CIs not including zero are highlighted in bold.

Similarly to the adults, the juveniles’ meta-analytic mean effect of anthropogenic habitats on the concentrations of bufadienolide compounds did not differ from zero (Table 2), but the effect sizes varied significantly by habitat type (moderator effect of habitat type, urban compared to agricultural: 0.42 ± 0.09, P < 0.001). However, the direction of these differences was opposite to what we found in adults. Compared to the toxin samples of juveniles originating from natural habitats, the overall concentration of bufadienolides tended to be lower in agricultural samples and higher in urban samples, although neither of these effects was significant (Table 2). In contrast with the adults, in the juveniles there was no significant interaction between habitat type and toxin type (P = 0.706; Table 2, Fig. 3), and juveniles from agricultural habitats had significantly lower concentrations of bufotoxins compared to their conspecifics originating from natural ponds (Table 2, Fig. 3). The above meta-analytic results were corroborated when we included additional moderators to enhance model fit (Table S7, Fig. S4) and when we re-categorized two juvenile compounds of uncertain toxin type (Table S8).

## Discussion

The parotoid is by far the largest toxin gland in toads, and larger parotoids contain more toxins^12,18^. Thus, our finding that adult toads had larger parotoids in urban and agricultural habitats than in natural habitats suggests that the total amount of stored toxins was higher in the animals captured from anthropogenic habitats. Furthermore, the overall amount of bufadienolides per unit dry mass of parotoid secretion was higher in adult toads from agricultural habitats, suggesting that their toxin secretion was more potent. These results parallel the earlier experimental findings on toad tadpoles that herbicide exposure increases their bufadienolide content^26^. Altogether, one potential explanation for these results is that chemical pollutants in anthropogenic habitats may enhance the production of bufadienolides, which may be either a non-adaptive constraint or an adaptive response. On the one hand, bufadienolides are synthesized from the same precursor as glucocorticoid stress hormones and steroid sex hormones, starting with the same initial steps^20^. Thus, elevated bufadienolide levels may be by-products of physiological stress and/or the endocrine-disrupting effects known to be elicited by many anthropogenic pollutants^26^. Notably, elevated levels of stress hormones have been found in aquatic amphibians in urban areas^39^. On the other hand, increasing the levels of bufadienolides might also be an adaptive response to chemical stressors, because bufadienolides may act as regulators of the amphibian skin’s ion transport^40^ which can be altered by several pollutants^41–43^. Both of these potential mechanisms can apply to the adult toads in our present study, because in a joint study we found that our ponds contained larger numbers and higher concentrations of endocrine-disrupting chemicals in anthropogenic habitats than in natural habitats^27^. Experimental studies are needed to test whether these chemicals or other pollutants affect bufadienolide levels by any of the two mechanisms proposed above.

However, pollution and physiological stress are not the only features of anthropogenic habitats that may contribute to the higher levels of toad toxins. Deterrence of predators is considered the main function of amphibian toxins, and experiments with newts showed that individuals can upregulate their chemical defences in response to higher predation risk perceived because of predator presence or repeated predatory attacks^17^. Although we have no data on predator densities at our study sites, ample literature shows that generally both urban and agricultural environments differ from natural habitats in the composition of predator fauna^44,45^. While larger, specialist predators typically avoid anthropogenic habitats, avian and mammalian generalist mesopredators like corvids, badgers, martens, racoons, skunks and foxes (all known as predators of toads) are often more abundant in urban habitats, as are domestic cats and dogs^44,46^. Similarly, some corvids^47^ and herons^48^ occur at higher densities in agricultural habitats, and some toad-eating snakes can spend up to 80% of their time in crop fields^49^. Therefore, toads living in anthropogenic habitats may perceive higher overall predation risk and may adjust their toxin levels accordingly. This scenario is supported by our results that bufagenins, the typically more toxic compounds^19,23^, were present in higher concentrations in the parotoid secretion of adult toads from urban as well as agricultural habitats, compared to their counterparts from natural habitats. This difference was particularly prominent for urban habitats (i.e. almost twice as large effect size as for agricultural habitats), where the increase in bufagenins seems to have come at the expense of bufotoxins. It is thus possible that urban toads invest more heavily into producing and storing higher amounts of the more potent compounds even at the price of having to deal with higher autotoxicity^18^. Alternatively, toads might not store the bufagenins but rather produce them upon gland discharge by enzymatically cutting the side chain off of the bufotoxins^18,19^. In this case, our urban toads may have had a faster machinery for this process, allowing them to respond more rapidly to a predator attack (i.e. a human squeezing their parotoids). Clarifying the avenues by which anthropogenic environments affect toad chemical defences will take more detailed studies on their toxin physiology.

Our common garden experiment showed that the differences found in adult toads were not retained in their offspring when the latter were raised in the lab under identical environmental conditions. When we compared the animals originating from anthropogenic and natural habitats, the significant differences seen in adults were either not present in the juveniles or even showed the opposite direction. In contrast with the larger parotoids and higher bufadienolide concentrations in adults from agricultural habitats, their offspring had smaller parotoids and lower bufotoxin concentrations. Similarly, the juveniles originating from urban habitats showed neither the larger parotoids nor the higher concentration of bufagenins and lower concentration of bufotoxins that we found in their parents. These results indicate that genetic or epigenetic changes are not likely to be responsible for the differences we observed in the adult toads’ chemical defences between anthropogenic and natural habitats. Cautionarily, we cannot exclude the possibility that the differences seen in the adults are genetically determined but are only expressed after maturity and/or during the breeding season. However, the juveniles had well developed gonads by the time of sampling and, in other toad species, parotoids acquire the morphological and histochemical characteristics of adults before sexual maturity^28^ and little if any seasonal variation has been found in parotoid size and toxin composition^29,50^. Also, we cannot rule out that some similarity in habitat effects between the two age groups might have been masked by the minor differences in the methods applied to adults and juveniles to measure parotoids and sample toxins. However, calliper and photo measurements yielded qualitatively identical results for juvenile parotoid size (Table 1, Table S6), and chemical analyses revealed an effect of retention time that was consistent between juveniles and adult females (with a similar but non-significant trend in males; Table S7) despite the fact that the toxin-sampling methods were not identical in the two age groups. This similarity in retention-time effects indicates that the disparity between adult and juvenile habitat differences in toxin composition is unlikely to be a methodological artefact (for more details, see Supplementary Results 2.3). Thus, had the same differences by habitat of origin been present in juveniles as in adults, it is likely that we would have been able to detect them (note that we did find differences between the offspring of toads from natural and anthropogenic habitats in several traits other than toxicity^27^). Therefore, our findings suggest that phenotypic plasticity at the level of individuals may play an important role in the enhanced chemical defences of toads living in anthropogenic habitats. This is intriguing, because out of the few studies that have so far investigated the mechanisms by which anthropogenic environments influence the animals’ physiological and behavioural responses to risk^51–53^ or chemical stress^54,55^, the majority suggested microevolution or other transgenerational effects. It is possible that anthropogenic environments exert complex selection forces on toads’ chemical defences because of spatio-temporal heterogeneity in pollution^27^ and predation risk^44^, which should then favour the evolution and maintenance of phenotypic plasticity^9^. Also, the potential for microevolution may be constrained if heritability is low. Further studies are thus needed to quantify heritability and plasticity of chemical defence in toads, as has been done with other adaptive inducible defences^56^.

A further important question is whether the elevated levels of chemical defence are costly in terms of fitness. Although experiments with common toad tadpoles did not reveal any cost of bufadienolide production^24,57^, the adults in our present study might have traded-off some resources for increased toxin synthesis. For example, if toads from anthropogenic habitats converted the common steroid precursor into bufadienolides at the expense of sex hormones^20^, they might have suffered reduced reproductive success. In line with this idea, we found significantly reduced offspring performance in our toads from anthropogenic habitats^27^, which might also explain the lower bufotoxin levels of juveniles from agricultural habitats. Furthermore, higher toxicity of toads may negatively affect other species too. For example, the invasive cane toad (*Rhinella marina*) has caused drastic mortality among Australian native predators that are highly sensitive to bufadienolides^13^. Thus, understanding how widespread the anthropogenic effects on toad toxicity are, and what mechanisms govern them, may prove important for the conservation of toads and their predators alike.

## Supplementary information

Supplementary Information

Supplementary Dataset

## Data Availability

The datasets used in this study are available as part of the Supplementary Material (Supplementary Dataset 1).
