# Supplementary material for: Toads phenotypically adjust their chemical defences to anthropogenic habitat change
Source: Sci Rep. 2019 Feb 28;9:3163. doi: 10.1038/s41598-019-39587-3 (PMC6395641; doi:10.1038/s41598-019-39587-3)
Supplement: Supplementary file 1 — Supplementary Information [file 41598_2019_39587_MOESM1_ESM.pdf]

**SUPPLEMENTARY INFORMATION**

Bókony V., Üveges B., Verebélyi V., Ujhegyi N., Móricz Á.M.:

**Toads phenotypically adjust their chemical defences to anthropogenic habitat change**

DOI: 10.1038/s41598-019-39587-3

## 1. Supplementary Methods

### *1.1. Capture sites*

In the spring of 2017 we studied 4 natural, 4 urban and 4 agricultural ponds in Hungary[27]. We verified this habitat categorization by geoinformatics measurements of land use around each pond (Fig. S1). We aimed to capture at least 10 pairs from each pond; however, because the spawning season of common toads is extremely short (a few days), we could not capture gravid females at one natural and one agricultural pond. For the present study we used the animals from 9 out of the 12 ponds (Table S1) for the following reasons. First, to prevent some habitat types from being represented by sex-biased samples, we excluded the two ponds from which we could not capture females. Second, to keep the number of ponds per habitat type constant, we did not sample toads from one urban pond (Pilisszentiván) which was relatively close (ca. 1 km) to another urban pond (Pilisvörösvár) and we managed to capture more toads from the latter.

**Table S1.** Land use characteristics of the nine capture sites in the present study.

| | | Proportion of landscape cover | | | | | | |
|---|---|---|---|---|---|---|---|---|
| Pond | Habitat type* | Arable fields | Pastures | Natural vegetation | Residential | Public, built | Roads | Rail-roads |
| János-tó | N | 0 | 0 | 0.987 | 0 | 0 | 0.012 | 0 |
| Szárazfarkas | N | 0 | 0 | 0.988 | 0 | 0 | 0.012 | 0 |
| Bajdázó | N | 0 | 0.022 | 0.970 | 0 | 0 | 0.024 | 0 |
| Göd | U | 0 | 0 | 0.248 | 0.431 | 0.033 | 0.053 | 0.011 |
| Pilisvörösvár | U | 0.004 | 0.024 | 0.270 | 0.531 | 0.083 | 0.077 | 0.014 |
| Pesthidegkút | U | 0.013 | 0 | 0.156 | 0.724 | 0.031 | 0.077 | 0 |
| Határrét | A | 0.484 | 0.137 | 0.284 | 0.070 | 0 | 0.026 | 0 |
| Anyácsapuszta | A | 0.802 | 0.051 | 0.145 | 0 | 0 | 0.007 | 0 |
| Perőcsény | A | 0.346 | 0.141 | 0.498 | 0 | 0 | 0.014 | 0 |

*Habitat types: N = natural, U = urban, A = agricultural.

**Figure S1.** Grouping of the capture sites along gradients of urban and agricultural land use. Symbol colour indicates our subjective habitat categorization of habitat type (green circles: natural, amber squares: agricultural, red triangles: urban). To verify our subjective categorization, we quantified land use in a 500-m wide buffer zone around each pond; the details of these geoinformatics measurements were described in an earlier paper[27]. In short, we measured the area of seven land-use categories: “natural” vegetation (e.g. woodlands, non-agricultural meadows), arable fields, pastures, residential areas, public built areas (e.g. commercial and industrial areas), roads with vehicular traffic, and railroads (see Table S1). Using these seven landscape variables we performed a principal component analysis (PCA), which yielded two axes with >1 eigenvalue, explaining 80.8% of variation in total; urban landscape areas loaded positively on the first axis whereas agricultural landscape areas loaded positively on the second axis. The PCA was performed with the 12 ponds investigated in our previously published study[27], but only the 9 ponds included in the present study are shown here.

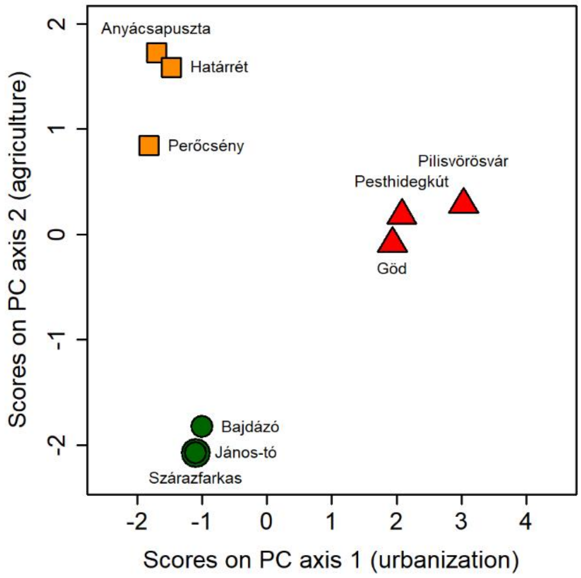


### *1.2. Animal handling and care*

Captured toads were transported to our laboratory in Budapest, where temperature was 20 ± 1.55 °C and artificial light-dark cycles mimicked the natural photoperiod. We housed each pair in a 52 × 37 × 33 cm plastic box filled with 15 L reconstituted soft water (RSW; 48 mg $NaHCO_3$, 30 mg $CaSO_4$ × 2 $H_2O$, 61 mg $MgSO_4$ × 7 $H_2O$, 2 mg KCl added to 1 L reverse-osmosis filtered, UV-sterilized tap water) and containing 4 vertical wooden sticks as spawning substrates. Each box housed one male and one female haphazardly chosen from the individuals captured at the same pond. The pairs were allowed to spawn for one week (they spawned 0-7 days after capture, with a median of 2 days; 87% spawned within 3 days), after which they were released along with most of their eggs at the pond where they had been captured.

From each pair, we kept ca. 30 eggs in the lab until hatching. When the embryos became free-swimming tadpoles, we selected 6 healthy-looking individuals (i.e. no visible deformities or abnormal behaviour) from each family and moved each into a 2-L plastic box filled with 1 L RSW; the remaining tadpoles were released to the pond where their parents had been captured. Out of every 6 siblings, 5 tadpoles were exposed to chemical treatments during their larval development as part of another experiment while one tadpole was kept in clean RSW as a control; only the latter were used in the present study. We raised the tadpoles to metamorphosis by feeding them with chopped spinach *ad libitum* and changing their rearing water twice a week. When a tadpole started metamorphosis (i.e. appearance of forelimbs), we decreased the water level to 0.1 L and slightly tilted the container to allow the animal to leave the water. When it completed metamorphosis (i.e. disappearance of the tail), we moved it into a clean rearing box containing wet paper towels as substrate and a piece of egg carton as shelter, which were changed every two weeks. Toadlets were fed ad libitum with springtails and small crickets, amended with a 3:1 mixture of $CaCO_3$ and Promotor 43 powder (Laboratorios Calier S.A., Barcelona, Spain) containing vitamins and amino acids. Juveniles were raised until ca. 5 months after metamorphosis (October 6 to November 10, 2017), which corresponds to the time of year when toads prepare for their first hibernation. The timing of photographing and euthanasia was balanced among the animals from the three habitat types such that natural, agricultural and urban individuals were systematically rotated during the one-month period. Photographing was done right after euthanasia in a standardized setting including a size reference as shown in Fig. S2.

### *1.3. Parotoid size measurements in toadlets*

From each photo, we measured SVL and the length and width of the left parotoid (in mm) using the ImageJ software[58]. Following earlier studies[28,29], we measured parotoid length as the longest straight line between the anterior and posterior end of the gland and parotoid width as the longest straight line perpendicular to parotoid length, measured roughly at the middle of the gland (Fig. S2); the same criteria as in adults (Fig. 1). We did not measure parotoid height as that would have required histological procedures[28].

All photo measurements had high within-observer repeatability (10 photos measured 9 times, on different days, by NU; Table S2). To explore the relationship between photo and calliper measurements, we also measured parotoid length and width by calliper on the toadlets' bodies that had been stored in ethanol for other purposes. These measurements were taken by another person (VB) who had also measured the parotoids of adult toads. Repeatability of calliper

measurements on the preserved juveniles was somewhat lower than the repeatability of photo measurements of live specimens (30 individuals measured 3 times, Table S2). The correlation between photo and calliper measurements was medium-strong for parotoid length ($r = 0.56$, $P < 0.001$, $n = 73$), width ($r = 0.46$, $P < 0.001$, $n = 73$) and area ($r = 0.61$, $P < 0.001$, $n = 73$). To understand the reasons for the imperfect agreement between the two kinds of measurements, we investigated whether the two observers taking each type of measurement differed when taking the same measurement. The 10 photos measured for repeatability by NU were re-measured once by VB, and we calculated the repeatability between the two observers by taking the average value of NU's 9 measurements for each photo. We found high between-observer repeatability for parotoid length (ICC = 0.934, 95% CI: 0.771, 0.983; $F_{9,10} = 29.3$, $P < 0.001$) but not for width (ICC = 0.159, 95% CI: -0.465, 0.691; $F_{9,10} = 1.38$ , $P = 0.311$) and area (ICC = 0.343, 95% CI: -0.298, 0.780; $F_{9,10} = 2.04$ , $P = 0.140$). Further inspection of the data revealed that there were two reasons for the low agreement on parotoid width. First, the width measurements of one observer were systematically smaller (by ca. 0.3 mm) than the other's. Second, in 2 out of the 10 animals, the parotoid had an irregular shape, making its borders difficult to identify and leading to relatively large differences (ca. 1.5 fold) between the two observers' measurements. Excluding these two animals, there was a strong correlation between the parotoid width measurements of the two observers ($r = 0.98$, $P < 0.001$, $n = 8$).

Based on these results, we decided to analyse the effects of habitat type on both kinds of parotoid measurements, because each method has its strengths and weaknesses. Photo measurements have high within-observer repeatability, but in some cases the pictures might be misleading because the full width of the parotoid might not always be completely visible. On the other hand, calliper measurements are somewhat less repeatable, but the borders of the parotoid and its widest width may be easier to identify when the animal is held in hand. Notably, we were blind regarding the animals' habitat of origin while taking both kinds of measurements, so the above sources of error are unlikely to introduce bias (rather than just noise) into our data. We decided to present the results on calliper measurements in the main text for greater consistency with the methods for adults, but we also present the results on photo measurements below (see 2.2 below).

**Figure S2.** Photograph of a captive-raised juvenile toad, used for measuring snout-vent length and parotoid size. Yellow lines show the length and width of the left parotoid gland.

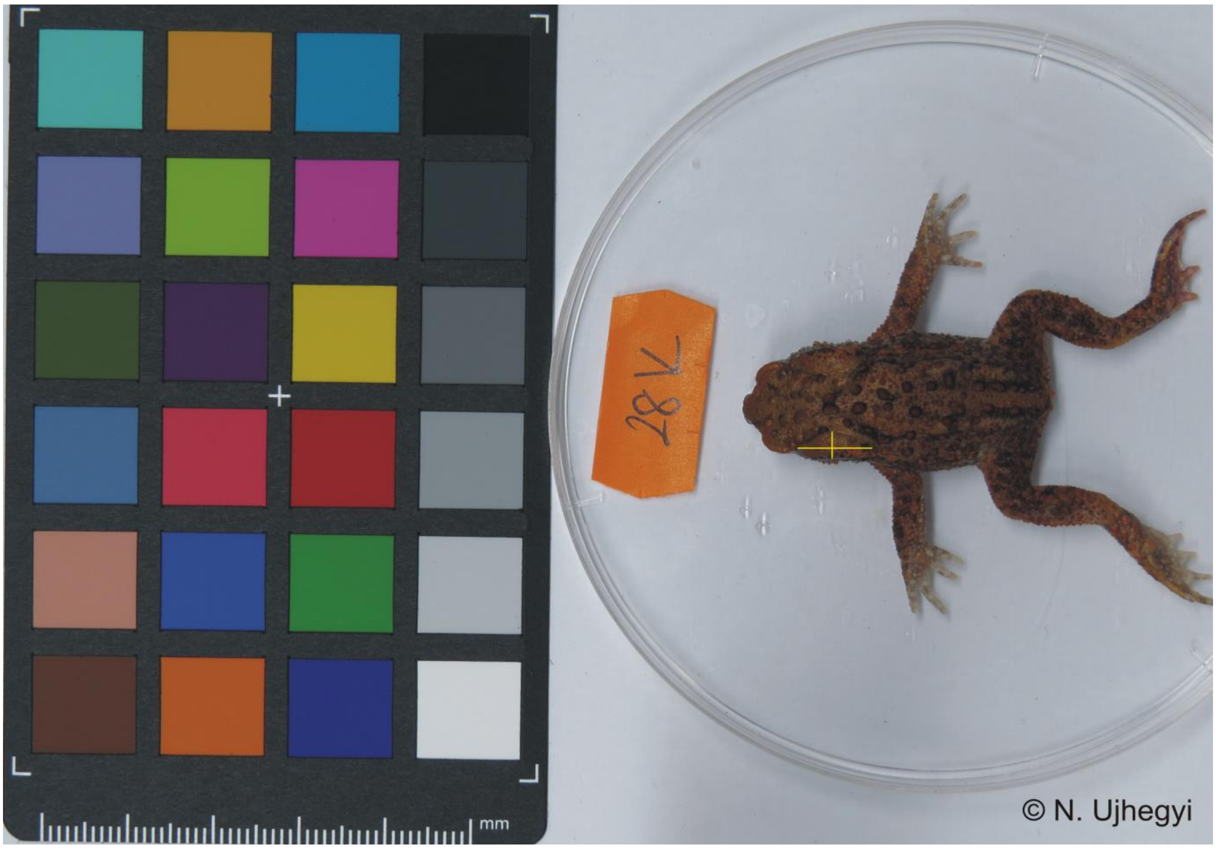


**Table S2.** Within-observer repeatability (intra-class correlation coefficient, ICC; with 95% confidence intervals, CI) of parotoid measurements taken from photos and with calliper.

| Method | Measurement | ICC | 95% CI | F | df | P |
|---|---|---|---|---|---|---|
| Photo | Snout-vent length | 0.997 | 0.993, 0.999 | 3116 | 9, 80 | <0.001 |
| | Parotoid length | 0.981 | 0.957, 0.994 | 457 | 9, 80 | <0.001 |
| | Parotoid width | 0.919 | 0.831, 0.975 | 103 | 9, 80 | <0.001 |
| | Parotoid area | 0.939 | 0.871, 0.982 | 140 | 9, 80 | <0.001 |
| Calliper | Parotoid length | 0.785 | 0.650, 0.882 | 12 | 29, 60 | <0.001 |
| | Parotoid width | 0.646 | 0.459, 0.796 | 6.5 | 29, 60 | <0.001 |
| | Parotoid area | 0.660 | 0.477, 0.805 | 6.82 | 29, 60 | <0.001 |

### *1.4. Chemical analysis of toxin samples*

All samples were stored at -20°C until chemical analysis. The toxin samples of adult toads were processed as follows. After a brief vortexing, we discarded the swab and filtered the remaining solution using nylon syringe filters with 0.22 µm pore size. From each sample, we took a 1-mL subsample and dried it at room temperature (ca. 22°C) under vacuum (SpeedVac ISS 100 concentrator, Savant Instruments Inc., Farmingdale, NY, USA) for ca. 3 hours. We measured its dry weight using a Sartorius Entris 224i-1S analytical balance (Sartorius AG, Göttingen, Germany) to the nearest 0.1 mg, and we re-dissolved it in 1 mL HPLC-grade absolute methanol. From each of these samples, we prepared a sample for analysis by adjusting the concentration to 0.1 mg/mL (0.1 mg dried secretion in 1 mL methanol). The parotoid samples of juveniles were homogenized, and dried under vacuum at 45°C using a Büchi Rotavapor R-134 rotary evaporator (Flawil, Switzerland). We measured dry weight as described above, re-dissolved each sample in 1 ml HPLC-grade absolute methanol (aided by exposing the samples briefly to ultrasound in a Tesla UC005AJ1 bath sonicator), and filtered the re-dissolved samples using nylon syringe filters with 0.22 µm pore size.

Quantitative measurement of bufadienolide compounds was carried out by a single-quadrupole HPLC-MS system (Model LC-MS-2020, Shimadzu, Kyoto, Japan) equipped with a binary gradient solvent pump, a vacuum degasser, a thermostated autosampler, a column oven, a photodiode detector and a mass analyser with electrospray ionization (ESI/MS). One µL of each adult sample and five µL of each juvenile sample was injected and analysed at 35°C on a Kinetex $C_{18}$ 2.6 µm column (100 mm × 3 mm i.d.) in series with an octadecyl $C_{18}$ guard column (4 mm × 3 mm i.d.). Eluent A was 5% aqueous acetonitrile with 0.05% formic acid and eluent B was acetonitrile with 0.05% formic acid. The flow rate was 0.6 mL/min and the gradient was as follows: 0-1 min, 10-20% B; 1-11 min, 20-29% B; 11-14 min, 29-70% B; 14-16 min, 100% B; 16-20 min, 10% B. ESI conditions were as follows: interface temperature, 350°C; desolvation line (DL) temperature, 250°C; heat block temperature, 400°C; drying $N_2$ gas flow, 15 L/min; nebulizer $N_2$ gas flow, 1.5 L/min; positive ionization mode. Full scan spectra in the range of m/z (mass-to-charge ratio) values 350–800 were recorded, and selected-ion monitoring (SIM) acquisition detecting the base peak of the bufadienolides we previously found in common toads[24,32] was performed as well. Bufadienolides were recognized by their characteristic UV spectra, and identified by comparing their peak retention time and m/z values to those of commercially purchased standards (all bufagenins: bufalin, bufotalin, resibufogenin, gamabufotalin, arenobufagin, telocinobufagin, cinobufotalin, digitoxigenin; note that standards for bufotoxins are not commercially available) and to the peaks present in a toxin sample

obtained from juvenile common toads[24,32]. When analysing the juveniles' samples, we also used a marinobufotoxin standard obtained as courtesy from Professor Robert Capon (University of Queensland, Australia).

The HPLC data were acquired and processed using the LabSolutions 5.72v software. We considered a compound to be present when its signal to noise ratio was at least 3 in the chromatogram. Because standards exist for some but not all bufadienolide compounds, we calculated approximate estimates of bufadienolide quantities using an approach that has been used in several previous studies[24–26,32,59,60]. For each bufadienolide compound in each sample, we estimated its quantity from the area values of SIM chromatogram peaks based on the calibration curve of the marinobufotoxin standard, and expressed its concentration as its marinobufotoxin-equivalent quantity per unit dry mass (ng/mg). We used the calibration curve of the marinobufotoxin standard because this was the only bufotoxin compound for which we had a standard, and the majority of compounds we found were bufotoxins (Table S3). Furthermore, the marinobufotoxin calibration curve had a medium slope among our standards (i.e. other standards had steeper or less steep calibration curves in our samples).

For the statistical analyses, bufadienolide compounds with m/z values below 500 and above 600 were categorized as bufagenins and bufotoxins, respectively. Although there exists a third type of bufadienolides, bufolipins, these compounds have only been identified from toad eggs and ovaries yet[61]. Their chemical structure is similar to bufotoxins', but the side chain on the sterane skeleton does not contain nitrogen. The molecular weight of bufolipins is ca. 630-660 Da, falling between those of bufagenins (ca. 400 Da) and bufotoxins (ca. 700 Da). Based on these data and on MS/MS analyses of bufadienolides previously extracted from common toad tadpoles, we are certain that in the present study we correctly categorized the compounds with <500 m/z as bufagenins. The smallest two compounds we categorized as bufotoxin had an m/z value of 627 and 629, respectively (found only in juveniles' parotoid samples). A similar compound with 627 m/z was isolated from tadpoles in our earlier studies, and MS/MS analysis suggested two molecular formulae with high likelihood: one did and one did not contain nitrogen. Therefore, we cannot exclude the possibility that "bufotoxin 1" and "bufotoxin 2" in Table S3 were bufolipins. However, this uncertainty did not influence the conclusions drawn from our statistical analyses (see Table S8). The next smallest compound we categorized as bufotoxin had an m/z value of 671; a similar compound isolated from tadpoles was very likely to contain nitrogen according to our MS/MS analysis. Nitrogen content was also supported by MS/MS analysis for several compounds with m/z values between 713 and 757. Thus, we can safely assume that the compounds with ≥671 m/z in our samples were bufotoxins.

**Table S3.** Bufadienolides found in the present study. Compounds with m/z (mass-to-charge ratio) values <500 and >600 were categorized as bufagenins and bufotoxins, respectively.

| | | Retention time (min) | | Occurrence (%) | |
|---|---|---|---|---|---|
| Compound | m/z | Adults | Juveniles | Adults | Juveniles |
| Bufagenin 1 (resibufogenin) | 385 | 14.4 | - | 95.2 | 0 |
| Bufagenin 2 (bufalin) | 387 | 13.6 | - | 97.0 | 0 |
| Bufagenin 3 (gamabufotalin) | 403 | 4.2 | - | 49.4 | 0 |
| Bufagenin 4 | 403 | 7.6 | - | 15.7 | 0 |
| Bufagenin 5 (telocinobufagin) | 403 | 9.8 | - | 96.4 | 0 |
| Bufagenin 6 | 415 | 7.4 | 6.7 | 98.2 | 100 |
| Bufagenin 7 (arenobufagin) | 417 | 5.6 | - | 47.0 | 0 |
| Bufagenin 8 | 417 | 6.1 | 5.6 | 99.4 | 100 |
| Bufagenin 9 | 419 | 3.7 | - | 97.6 | 0 |
| Bufagenin 10 (bufotalin) | 445 | 10.8 | 9.9 | 99.4 | 100 |
| Bufagenin 11 | 453 | 4.1 | - | 99.4 | 0 |
| Bufagenin 12 | 453 | - | 2.5 | 0 | 100 |
| Bufotoxin 1 | 627 | - | 10.8 | 0 | 99 |
| Bufotoxin 2 | 629 | - | 9.6 | 0 | 99 |
| Bufotoxin 3 | 671 | 12.1 | 11.2 | 96.4 | 100 |
| Bufotoxin 4 | 671 | - | 13.0 | 0 | 100 |
| Bufotoxin 5 | 679 | - | 5.2 | 0 | 100 |
| Bufotoxin 6 | 683 | - | 8.6 | 0 | 100 |
| Bufotoxin 7 | 685 | - | 7.3 | 0 | 68 |
| Bufotoxin 8 | 687 | 4.8 | - | 55.4 | 0 |
| Bufotoxin 9 | 697 | 14.0 | - | 81.9 | 0 |
| Bufotoxin 10 | 699 | 13.7 | - | 96.4 | 0 |
| Bufotoxin 11 | 701 | 3.3 | - | 99.4 | 0 |
| Bufotoxin 12 | 701 | 5.0 | - | 86.7 | 0 |
| Bufotoxin 13 | 701 | 6.0 | - | 48.2 | 0 |
| Bufotoxin 14 | 701 | 8.7 | - | 94.6 | 0 |
| Bufotoxin 15 | 713 | 6.9 | - | 98.8 | 0 |
| Bufotoxin 16 (marinobufotoxin) | 713 | 11.9 | 10.9 | 99.4 | 100 |
| Bufotoxin 17 | 715 | 6.3 | 5.5 | 98.8 | 100 |
| Bufotoxin 18 | 715 | 7.9 | - | 46.4 | 0 |
| Bufotoxin 19 | 715 | 10.6 | 9.8 | 99.4 | 100 |
| Bufotoxin 20 | 715 | - | 11.5 | 0 | 100 |
| Bufotoxin 21 | 727 | 8.5 | 7.7 | 99.4 | 100 |
| Bufotoxin 22 | 729 | 7.6 | 6.8 | 100 | 100 |
| Bufotoxin 23 | 729 | 9.7 | - | 48.8 | 0 |
| Bufotoxin 24 | 729 | 9.9 | 9.1 | 99.4 | 100 |
| Bufotoxin 25 | 731 | 6.9 | - | 95.8 | 0 |
| Bufotoxin 26 | 743 | 12.1 | 11.1 | 99.4 | 100 |
| Bufotoxin 27 | 757 | 13.4 | 13.1 | 100 | 100 |

## 2. Supplementary Results

### *2.1. Finding the best modelling structure*

To test if site of origin was a significant random effect, for each dependent variable we compared the fit of two models, one with and one without site as a random intercept, using likelihood-ratio tests[36,62]. For parotoid area, the model with site as a random factor was a linear mixed-effects (LME) model including the fixed effects of sex, SVL, their interaction, and habitat type, whereas the model without site as a random factor was a generalized least squares (GLS) model including the same fixed effects.

To analyse toxin composition, we included all bufadienolide compounds in a single model (one for adults, one for juveniles). For each age group, we compared two LME models that both contained individual as a random factor, and one also included site (with individual nested within site). In both models, the fixed effects were compound, sex, habitat type, all their two-way interactions and their three-way interaction. Because there were large differences among compounds in their quantities' range, and some compounds were not detected (i.e. had zero quantity) in some individuals, we transformed the bufadienolide quantities as $\log_{10}$(value + the lowest non-zero value across all samples in that age group) to ensure that the distribution of residuals does not violate the LME models' requirements, and we allowed for heteroscedasticity using the "varIdent" variance structure[36].

In all cases, site as a random factor did not improve model fit significantly (Table S4). Therefore we decided not to include site in subsequent analyses. Also, the results of these models yielded further insights for finding the best statistical approach for analysing toxin composition. Specifically, the LME models including all bufadienolide compounds showed that the compound × habitat type interaction was highly significant for both adults and juveniles (Table S5). This means that the differences between habitats vary among compounds, so we decided to quantify the habitat effects separately for each compound and then use meta-analysis for quantifying the overall effects of each habitat type and the effects of moderator variables. For adults, the three-way interaction of sex × compound × habitat type was also significant (Table S5), whereas for juveniles, neither the main effect of sex nor any of its interactions was significant (Table S5). These findings validate our approach that we calculated the effect sizes for the meta-analyses separately for the two sexes in adults but we pooled males and females in juveniles.

**Table S4.** Results of likelihood-ratio tests comparing models with and without site of origin as a random intercept.

| Dependent variable | AIC when site as random factor included | excluded | ΔAIC | P |
|---|---|---|---|---|
| Parotoid area | | | | |
| Adults | 1416.46 | 1417.04 | 0.58 | 0.109 |
| Juveniles | | | | |
| Calliper | 355.87 | 353.87 | 2.00 | 0.999 |
| Photo | 338.91 | 340.72 | 1.82 | 0.051 |
| Bufadienolide concentration | | | | |
| Adults | 1761.36 | 1762.19 | 0.83 | 0.092 |
| Juveniles | 93.84 | 91.79 | 2.05 | 0.829 |

**Table S5.** Type-2 ANOVA tables of the LME models of bufadienolide concentrations, with individual as random factor. Models also including site as random factor yielded qualitatively identical, quantitatively similar results (not shown).

| | Adults | | | Juveniles | | |
|---|---|---|---|---|---|---|
| Model terms | $\chi^2$ | df | P | $\chi^2$ | df | P |
| Compound | 57016.8 | 30 | <0.001 | 44940.9 | 19 | <0.001 |
| Habitat | 4.3 | 2 | 0.115 | 6.7 | 2 | 0.036 |
| Sex | 103.1 | 1 | <0.001 | 0.6 | 1 | 0.421 |
| Compound × Habitat | 305.3 | 60 | <0.001 | 130.4 | 38 | <0.001 |
| Compound × Sex | 8884.5 | 30 | <0.001 | 15.0 | 19 | 0.721 |
| Habitat × Sex | 3.7 | 2 | 0.155 | 2.5 | 2 | 0.283 |
| Compound × Habitat × Sex | 125.4 | 60 | <0.001 | 41.2 | 38 | 0.334 |

### *2.2. Parotoid size in juveniles measured from photos*

In agreement with the calliper measurements (Table 1), the photo measurements showed that the toadlets originating from agricultural habitats had significantly smaller parotoids than toadlets originating from natural habitats (Table S6). For toadlets from urban habitats, the results were also qualitatively similar with both kinds of measurements: there were no significant differences in parotoid size between toadlets originating from urban and natural habitats (Table S6). While the effect of urban habitat was positive and close to zero when using calliper measurements (Table 1), we found a small negative effect with photo measurements, which was nevertheless non-significant (Table S6).

**Table S6.** Parameter estimates of the linear model testing the effects of habitat type on juvenile parotoid size measured from photos (n = 73 toadlets, residual df = 67). Significant habitat differences are highlighted in bold.

| Model parameters | Estimate | SE | t | P |
|---|---|---|---|---|
| Intercept (natural, male) | 17.953 | 0.509 | 35.28 | <0.001 |
| SVL (mm, mean-centered) | 0.280 | 0.192 | 1.46 | 0.149 |
| Sex (female) | 1.113 | 0.571 | 1.95 | 0.056 |
| **Habitat (agricultural)** | **-2.309** | **0.771** | **-3.00** | **0.004** |
| Habitat (urban) | -1.111 | 0.727 | -1.53 | 0.131 |
| Sex × SVL | 0.634 | 0.245 | 2.59 | 0.012 |

### *2.3. Meta-analyses of bufadienolide concentrations*

The distribution of raw data used for calculating effect sizes are shown in Figure S3. Altogether, we found 39 bufadienolide compounds, 12 of which were detected in adults as well as juveniles; 19 compounds occurred only in adults and 8 only in juveniles (Table S3). This somewhat lower diversity of juvenile toxin samples might have been due to ontogenetic or other biological differences between adults and juveniles, or some compounds might have leached from the juvenile parotoids into the ethanol during storage. However, the compounds missing from juveniles typically occurred in low concentrations in adults (mean ± SE: 810 ± 19 ng/mg, compared to 8666 ± 248 ng/mg for compounds that were detected in both age groups; Fig. S3), so it is possible that they were below our detection limit in the small juvenile samples. Whether these compounds were truly absent in juvenile parotoids or went undetected in our HPLC analyses, it cannot account for our finding that juveniles did not show the same habitat effects as their parents did, because the compounds missing from juveniles showed similar habitat differences in adults as the compounds detected in both age groups (Fig. S3). Furthermore, the effect sizes varied with retention time (see below), yet the compounds we detected in adults but not in juveniles had both short and long retention times (ranging from 3.3 to 14.4 min; Table S3), and the retention times of compounds found in adults (mean ± SE: 8.6 ± 0.6) did not differ significantly from the retention times of compounds found in juveniles (mean ± SE: 8.8 ± 0.6; Welch test: $t_{45.6} = 0.22$, $P = 0.825$).

We explored the moderator effects of sex (in adults) and retention time (RT, related to the hydrophobicity of toxin compounds) to see if the results of our simpler meta-analyses hold in more complex models with lower heterogeneity and better model fit. For the adults, we built several meta-analyses that all contained the interaction of habitat type and toxin type, and we added sex and/or RT either as main effects or in interaction with each other or with habitat type or toxin type. Based on the models' $AIC_c$ values (Akaike's information criterion corrected for sample size), the best supported model had very strong support ($\Delta AIC_c > 20$) and included the interaction of sex and RT in addition to the interaction of habitat type and toxin type (Table S7). This model showed that the effect sizes decreased significantly with RT in females but not in males (Table S7, Fig. S4): compared to females from natural habitats, females from anthropogenic habitats tended to have higher concentrations of bufadienolides with short RT (relatively low hydrophobicity) and lower concentrations of bufadienolides with long RT (relatively high hydrophobicity). The interaction of habitat type and toxin type remained significant in this model (Table S7): compared to toads from natural habitats, urban males and females had more bufagenins and less bufotoxins, whereas toads from agricultural habitats had

higher concentrations of both types of bufadienolides, although the latter was significant only in males (Table S7). These effects were similar to those estimated without accounting for sex and RT (Table 2).

For the juveniles, we only considered RT as an additional moderator. However, because the interaction of habitat type and toxin type was non-significant (Table 2), we also evaluated models that excluded this interaction, or even the main effect of toxin type. The two best-fitting models ($\Delta AIC_c > 5$) included habitat type as a main effect and RT either as a main effect or in interaction with habitat type. Because these two models received very similar support ($\Delta AIC_c$ = 0.65), we preferred the simpler model, without interaction. This model showed that the effect sizes decreased significantly with RT (Table S7, Fig. S4), and that toadlets with urban origin had higher concentrations whereas toadlets with agricultural origin had lower concentrations of bufadienolides compared to toadlets originating from natural ponds (Table S7). The latter effects were similar to those estimated without including RT (Table 2), but had narrower confidence intervals and thus were significant (Table S7).

In the parotoid samples of juveniles, we found two bufadienolides that may be bufolipins (see 1.4 above). Because the toxicity of bufolipins is similar to bufagenins'[61], we repeated our meta-analysis by grouping the two compounds with 627 and 629 m/z together with bufagenins (instead of bufotoxins). Our results were qualitatively unchanged (Table S8): the interaction of habitat type and toxin type was still non-significant ($P = 0.661$), and the effect size estimates for each group were similar to what we report in the main text (Table 2).

**Table S7**. Estimates from the best-fitting meta-analytic models for adult and juvenile toads' toxin composition. Meta-analytic means (Hedges' d) are given as standardized differences in the concentrations of bufadienolide compounds between anthropogenic and natural habitats, with 95% confidence intervals (CI; in brackets). CIs not including zero are highlighted in bold. Effect sizes for retention time refer to the slope of relationship whereas the other effect sizes refer to the difference between anthropogenic and natural habitats at average retention time.

| Moderators | Adults | Juveniles |
|---|---|---|
| Urban habitats | | **0.184 (0.012, 0.356)** |
| Bufotoxins | | |
| Males | **-0.245 (-0.362, -0.128)** | |
| Females | **-0.420 (-0.532, -0.308)** | |
| Bufagenins | | |
| Males | **0.430 (0.275, 0.585)** | |
| Females | **0.256 (0.104, 0.408)** | |
| Agricultural habitats | | **-0.237 (-0.423, -0.051)** |
| Bufotoxins | | |
| Males | **0.220 (0.099, 0.341)** | |
| Females | 0.045 (-0.069, 0.160) | |
| Bufagenins | | |
| Males | **0.258 (0.097, 0.419)** | |
| Females | 0.083 (-0.073, 0.240) | |
| Retention time | | **-0.131 (-0.188, -0.075)** |
| Males | -0.008 (-0.034, 0.018) | |
| Females | **-0.085 (-0.109, -0.061)** | |

**Table S8**. Effects of anthropogenic habitats on toxin composition in captive-reared juvenile toads, when two compounds with 627 and 629 m/z value were categorized as bufagenins. Meta-analytic means (Hedges' d) are given with 95% confidence intervals (CI; in brackets); the CI not including zero is highlighted in bold.

| Habitat of origin | Toxin type | Effect (95% CI) |
|---|---|---|
| Urban | Bufotoxins | 0.085 (-0.192, 0.362) |
| | Bufagenins | 0.417 (-0.007, 0.8417) |
| Agricultural | Bufotoxins | **-0.309 (-0.599, -0.020)** |
| | Bufagenins | -0.066 (-0.508, 0.375) |

**Figure S3.** Concentration of each bufadienolide compound in toads originating from natural (N), agricultural (A) and urban (U) habitats. Compounds are named as in Table S3; asterisks mark the compounds that were found in both adults and juveniles. Note that the range of the Y axis varies between panels, and the panels are ordered from bottom to top. In each box plot, the filled dot is the median, the box is the interquartile range, the whiskers extend to the upper and lower quartile ± 1.5 × interquartile range, and open circles represent extreme data points. Only the compounds used in the meta-analyses are shown.

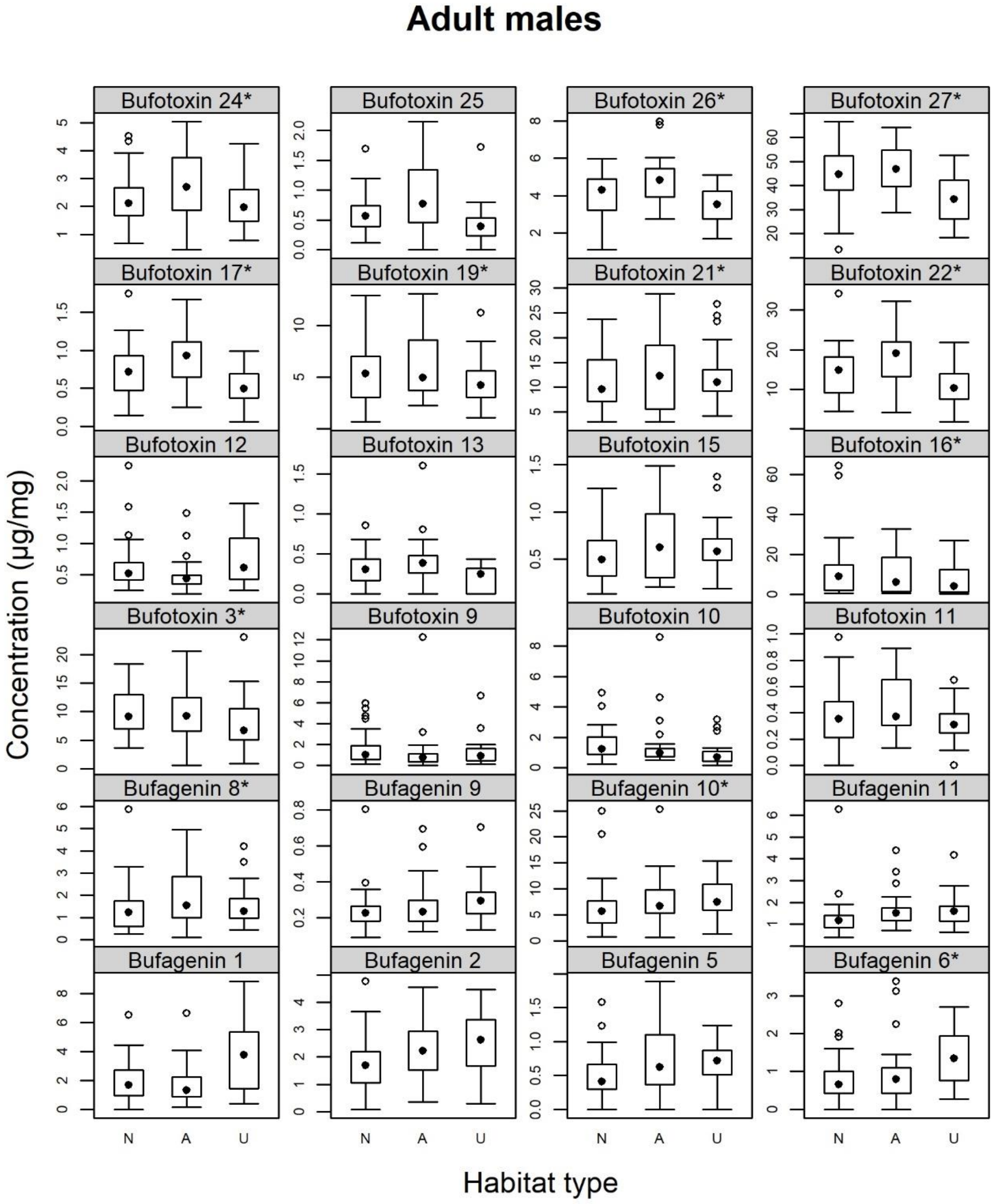

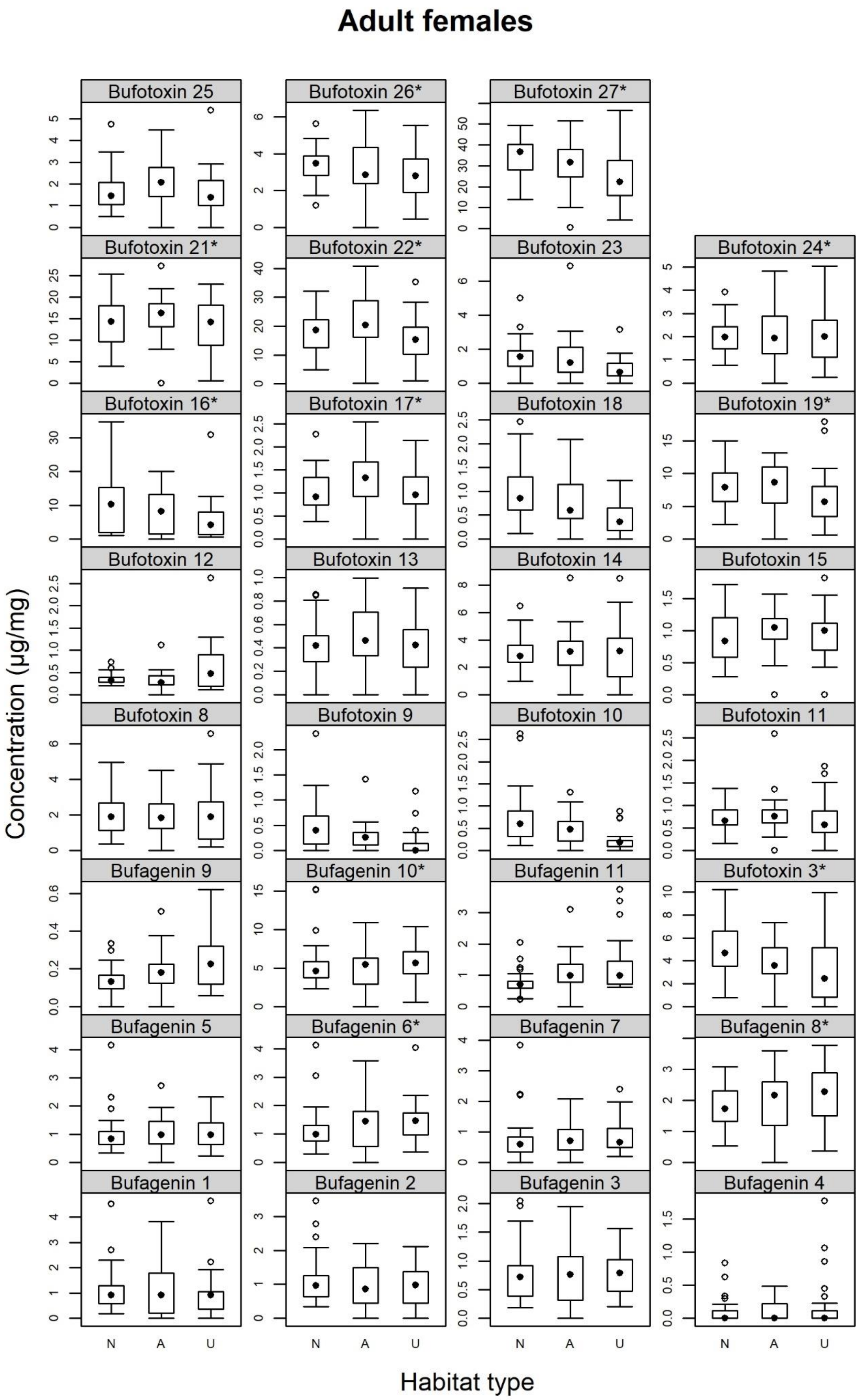
Adult females
Bufotoxin 25
Bufotoxin 26*
Bufotoxin 27*
Bufotoxin 21*
Bufotoxin 22*
Bufotoxin 23
Bufotoxin 24*
Bufotoxin 16*
Bufotoxin 17*
Bufotoxin 18
Bufotoxin 19*
Bufotoxin 12
Bufotoxin 13
Bufotoxin 14
Bufotoxin 15
Bufotoxin 8
Bufotoxin 9
Bufotoxin 10
Bufotoxin 11
Bufagenin 9
Bufagenin 10*
Bufagenin 11
Bufotoxin 3*
Bufagenin 5
Bufagenin 6*
Bufagenin 7
Bufagenin 8*
Bufagenin 1
Bufagenin 2
Bufagenin 3
Bufagenin 4
Concentration (µg/mg)
N A U
Habitat type

# Juveniles

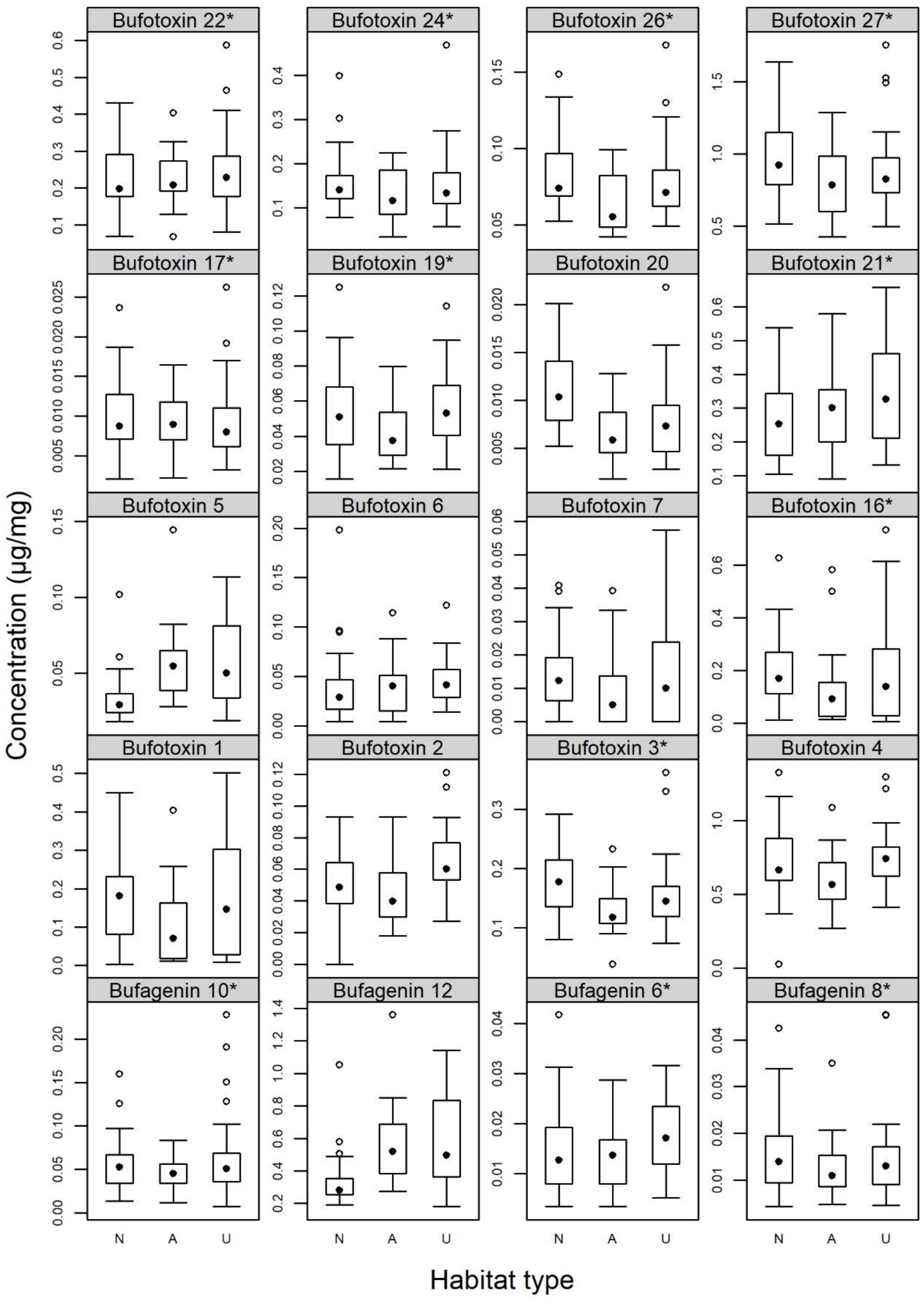

Bufotoxin 22*
Bufotoxin 24*
Bufotoxin 26*
Bufotoxin 27*
Bufotoxin 17*
Bufotoxin 19*
Bufotoxin 20
Bufotoxin 21*
Bufotoxin 5
Bufotoxin 6
Bufotoxin 7
Bufotoxin 16*
Bufotoxin 1
Bufotoxin 2
Bufotoxin 3*
Bufotoxin 4
Bufagenin 10*
Bufagenin 12
Bufagenin 6*
Bufagenin 8*
Concentration (µg/mg)
N
A
U
Habitat type

**Figure S4**. Effects of anthropogenic habitats on the concentrations of bufadienolide compounds, in relation to the compounds' retention time in HPLC analysis. Higher retention times correspond to higher hydrophobicity. Error bars represent the meta-analytic means with 95% confidence intervals (CI), i.e. the standardized differences between natural and anthropogenic habitats in the animals' bufadienolide concentrations (amount of each compound per unit dry mass of toxin sample).

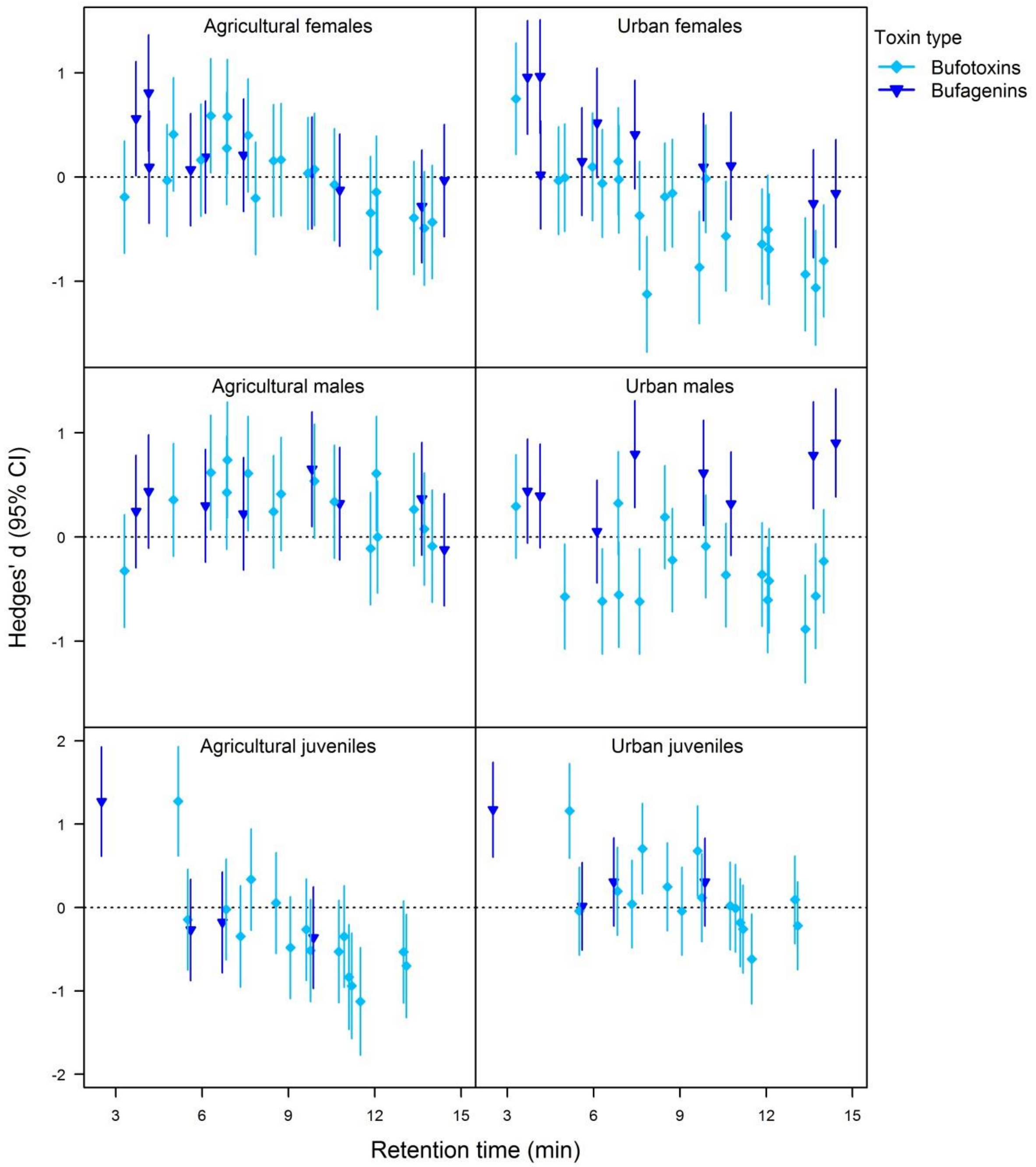

### 3. Supplementary References


58. Schneider, C. A., Rasband, W. S. & Eliceiri, K. W. NIH Image to ImageJ: 25 years of image analysis. Nat. Methods 9, 671–675 (2012).

59. Benard, M. F. & Fordyce, J. A. Are induced defenses costly? Consequences of predator-induced defenses in western toads, Bufo boreas. Ecology 84, 68–78 (2003).

60. Hagman, M., Hayes, R. A., Capon, R. J. & Shine, R. Alarm cues experienced by cane toad tadpoles affect post-metamorphic morphology and chemical defences. Funct. Ecol. 23, 126–132 (2009).

61. Matsukawa, M. et al. Isolation and characterization of novel endogenous digitalis-like factors in the ovary of the giant toad, Bufo marinus. J. Nat. Prod. 61, 1476–1481 (1998).

62. Bolker, B. M. et al. Generalized linear mixed models: a practical guide for ecology and evolution. Trends Ecol. Evol. 24, 127–135 (2009).
